# Regulation of carcinogenesis through multiple zinc fingers in ZBTB20†

**DOI:** 10.1039/d5cb00088b

**Published:** 2025-06-09

**Authors:** Hyunyong Kim, Yunha Hwang, Jin Sung Cheong, Seung Jae Lee

**Affiliations:** a Department of Chemistry, Jeonbuk National University Jeonju 54896 Republic of Korea slee026@jbnu.ac.kr; b Department of Neurology, Wonkwang University Hospital Iksan 54538 Republic of Korea; c Research Institute for Molecular Biology and Genetics, Jeonbuk National University Jeonju 54896 Republic of Korea

## Abstract

Zinc finger (ZF) proteins regulate transcription by interacting with *cis*-acting elements for gene expression in response to stimuli within physiological systems. Bioinformatic studies have proposed that zinc finger and BTB (Broad complex, Tramtrack, Bric-à-brac) domain-containing protein 20 (ZBTB20) acts as a key regulator of crucial genes associated with carcinogenesis. However, biochemical experiments using purified proteins remain unreported. In this study, we investigated the regulatory functions of the ZF domains in ZBTB20, which has five CX_2_CX_12_HX_3_H-type classical ZF domains, in the inhibition and expression of downstream transcription factors, including alpha-fetoprotein (AFP) and forkhead box transcription factor O1 (FOXO1). The four ZF domains of ZBTB20, ZBTB20(ZF1-4), inhibit the expression of AFP with specificity (*K*_d_ = 0.6 ± 0.04 nM) by interacting with the *afp* promoter (5′-ACCTA). Furthermore, ZBTB20(ZF1-4) or ZBTB20(ZF1-5) inhibited the expression of FOXO1, thereby suppressing cell cycle arrest and inducing tumorigenesis by binding to the promoter of *foxO1* (5′-ACCGCCGCCTC) with affinities of 1.7 ± 0.07 and 2.1 ± 0.05 nM, respectively. The results provide the first evidence that ZBTB20 regulates gene expression through ZF domains positioned at the C-terminus through interactions with *cis*-acting elements to achieve specificity and selectivity. The balance of ZBTB20 expression can be a crucial issue for the regulation of two downstream transcription factors to maintain homeostasis.

## Abstract 2

ZBTB20 binds to the *afp* and *foxO1* promoters, represses their transcription, and regulates hepatocarcinogenesis.

## Graphical abstract

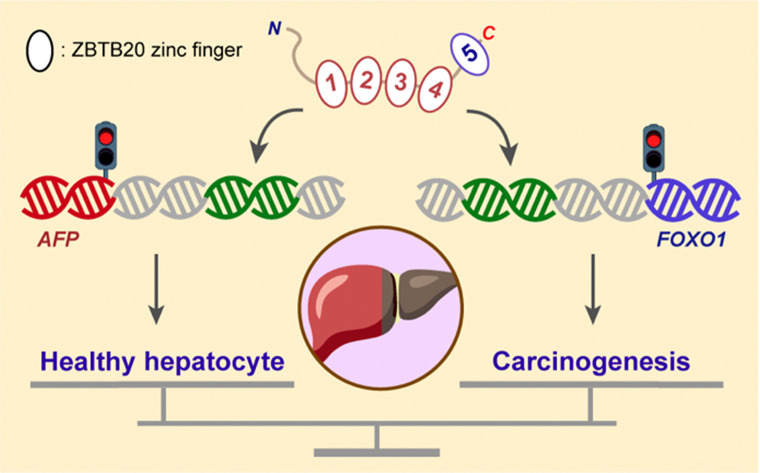

## Introduction

**Fig. 1:**
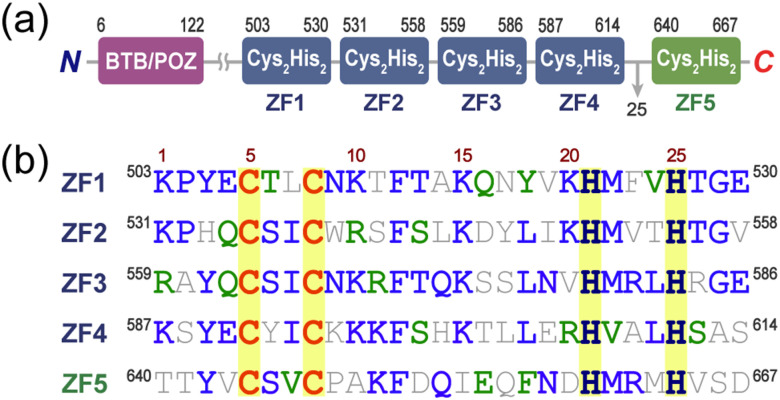
ZBTB20 from *Homo sapiens*. (a) Domain organization of ZBTB20. The *N*-terminal BTB/POZ domain is followed by five classical ZF domains (ZF1–ZF5). The linker region between ZF4 and ZF5 comprises 25 amino acids, which is longer than the typical linkers in classical ZF proteins. (b) Sequence alignment of five classical ZF domains in ZBTB20. Each color denotes an identity (blue), similarity (green), and mismatch (gray) sequence. Yellow highlights indicate cysteine (Cys) and histidine (His) residues involved in zinc ion coordination, characteristic of the CX_2_CX_12_HX_3_H-type zinc finger motif.

The central dogma is precisely controlled to respond to internal and external stimuli while maintaining homeostasis within the biological system.^1,2^ Zinc finger (ZF) proteins, characterized by one or more ZF domains within a single polypeptide chain, are among the most common regulators of these biological processes.^3,4^ Recent studies have suggested that ZBTB20 (zinc finger and BTB domain-containing protein 20) is associated with carcinogenesis, including hepatocellular carcinoma (HCC), non-small cell lung cancer (NSCLC), breast cancer, gastric adenocarcinoma, and acute myeloid leukemia.^5–9^ Studies on ZBTB20 knockout mice have demonstrated that the absence of this protein results in growth retardation, disrupted hippocampal development, abnormal hormonal responses, and impaired glucose metabolism, with the test animals unable to survive beyond 12 weeks.^10–13^ The expression level of ZBTB20 regulates developmental and differentiation pathways by interacting with *cis*-acting elements involved in specific gene expression.^14–16^ ZBTB20 comprises a Broad complex, Tramtrack, Bric-à-brac (BTB) domain at the N-terminus, which induces the formation of homodimeric or oligomeric complexes, and five ZF domains located in the C-terminal region (Fig. 1a and Fig. S1, ESI†).^10,17^ ZBTB20 belongs to the CX_2_CX_12_HX_3_H-type ZF protein family, which includes parkin interacting substrate (PARIS) and zinc finger 18 (ZNF18), and is expressed in most organs, notably the brain.^18^ PARIS contains four ZF domains and fluorescence anisotropy studies have demonstrated that three classical ZF domains, PARIS(ZF2-4), repress dopamine secretion by interacting with the promoter region of peroxisome proliferator-activated receptor (PPAR) gamma coactivator-1 alpha (PGC-1α).^19,20^ ZNF18 has five ZF domains in the C-terminal region, and bioinformatic studies have suggested that these ZFs interact with the promoter elements of cyclin-dependent kinase 1 (CDK1) to inhibit gene expression.^21,22^ Further research is needed to elucidate the functions of the five ZF domains of ZBTB20 in specific gene regulation (Fig. 1b).

ZBTB20 null mice exhibit severely impaired glucose metabolism and homeostasis due to impaired liver function.^10^ Previous studies have suggested that alpha-fetoprotein (AFP), a member of the albuminoid gene family in the liver, is directly repressed by ZBTB20, a sequence-specific transcriptional repressor.^23^ AFP serves as the primary tumor marker and is extensively applied for screening and diagnosing HCC because of its active role in tumor progression.^24,25^ HCC is the most common type of primary liver cancer associated with chronic liver disease, and early detection is crucial for improving survival rates.^26^ AFP plays a vital role in fetal survival and during the perinatal period by transporting molecules, including metal ions, fatty acids, medications, and bilirubin (Fig. S2a, ESI†).^27,28^ The expression of AFP is regulated by the hepatocyte nuclear factor (HNF) family and CCAAT/enhancer binding protein (C/EBP), although the level of AFP is completely diminished (<10 ng mL^−1^) within a few weeks after birth.^29,30^ This downregulation is mediated by two ZF proteins, zinc fingers and homeoboxes 2 (ZHX2) and ZBTB20.^14,31^ ZHX2 suppresses the activity of hepatic nuclear factor 1 (HNF1), which binds to the *afp* promoter region to retard transcriptional activities, whereas ZBTB20 directly interacts with specific sequences of the *afp* promoter.^23,31^ Clinical studies have demonstrated that elevated AFP levels are indicative of tumor aggressiveness, with anti-apoptotic effects mediated through TNF-related apoptosis-inducing ligand (TRAIL) and paclitaxel chemotherapy.^32^ Genetic studies on mouse AFP have demonstrated that the unique binding sites of ZBTB20 are highly conserved in humans and are specific to the liver, however, dispensable in other tissues.^23^ These studies demonstrate the role of ZBTB20 in AFP regulation, although further investigation is needed to understand the specific transcription factors and elements involved.

Forkhead box transcription factor O1 (FOXO1) is considered a tumor suppressor in HCC, as it directly targets specific cancer cells and alters the immunogenicity of the tumor microenvironment.^33^ As a member of the FOX superfamily, FOXO1 has a wide range of functions, as it modulates downstream targets associated with apoptosis genes, cell cycle arrest genes, and metabolic and immune regulators.^34–37^ It comprises four functional domains: the nuclear localization signal, nuclear export signal, transactivation domain, and forkhead (FKH) domains.^38^ Helix 3 in the FKH domain identifies specific DNA sequences, such as the Daf-16 element (5′-GTAA(T/C)) or insulin response sequence (IRS, 5′-(C/A)(A/C)AAA(C/T)AA) (Fig. S2b, ESI†).^39,40^ FOXO1 expression is meticulously regulated by multiple transcription factors in response to internal and external stimuli to maintain cellular homeostasis.^41,42^ The prominent role of FOXO1 makes it a crucial target for protein-based therapeutics because of its involvement in apoptosis and cell cycle arrest.^43^ FOXO1 initiates substantial tumorigenesis inhibition by enhancing the expression of B-Cell Lymphoma 2-like protein (BCL-2) interacting mediator (BIM) and growth arrest and DNA damage-inducible protein 45 (GADD45), activating apoptotic cascades and cell cycle arrest regulators.^38^ Research on the relationship between ZBTB20 and FOXO1 has been investigated in HCC cell lines, including Hep3B, Huh7, HepG2, and SMMC-7721 which have shown that the mRNA and protein levels of ZBTB20 are elevated in HCC compared to normal liver cells.^33^ ZBTB20 knockout mice exhibit increased mRNA and protein levels of FOXO1, which inhibits cell proliferation.^6^ The promoter region (−1000 to +10 bp) of the *foxO1* gene was examined to identify the interaction region of ZBTB20, and chromatin immunoprecipitation suggested that ZBTB20 interacts at the −200 to −100 bp promoter region.^33^ Systematic approaches to understand the interaction details between ZBTB20 and *foxO1* can provide valuable information to address the challenges of HCC.

The specifics of ZBTB20 do not provide direct evidence of its transcriptional regulation of the two key transcription factors, AFP and FOXO1, in HCC, although ZF domains are potential candidates for these interactions.^23,33^ Notably, the arrangement of ZF domains shows that the first four ZF domains, ZBTB20(ZF1-4), are linked to the 5th ZF domain by 25 amino acids (Fig. 1a). KRAB-containing classical ZFs typically have a short TGERP linker, whereas ZBTB20(ZF1-5) lacks these traditional linker patterns between the 4th and 5th ZF domains.^44,45^ These results suggest that ZBTB20(ZF1-4) and ZBTB20(ZF5) do not share the same binding partner. The biophysical interactions of ZBTB20(ZF1-4) and ZBTB20(ZF1-5) were investigated in relation to their binding partners, including *afp* and *foxO1* promoters. This experimental plan aimed to directly address two questions with detailed evidence: (1) the functional roles of ZF domains in ZBTB20 and (2) the critical sequence information of *foxO1* and *afp* for ZBTB20 interactions. The balancing effects of ZBTB20 can be explained when the *K*_d_s between ZBTB20 and the promoters of *foxO1* or *afp* are identified. ZBTB20 inhibits HCC by inhibiting AFP and induces HCC by inhibiting FOXO1.^23,33^ In this study, we examined the *K*_d_s, specific sequences, and metal interferences to scrutinize these binding events through overexpressed and purified Zn^2+^–ZBTB20(ZF1-4) and Zn^2+^–ZBTB20(ZF1-5). These biochemical studies with purified ZF domains from ZBTB20 provide insights into the binding between ZF domains and nucleic acids. Furthermore, the outcomes of these studies can assist in the design of promising protein therapeutics for the treatment and diagnosis of HCC.

## Results and discussion

### Expression and purification of ZBTB20(ZF1-4) and ZBTB20(ZF1-5)

Codon-optimized versions of ZBTB20(ZF1-4, 118 amino acids, 13 913.21 Da) and ZBTB20(ZF1-5, 169 amino acids, 19 196.16 Da) were overexpressed in *Escherichia coli* (*E. coli*) and purified using a two-step procedure to achieve a purity level exceeding 95% (Fig. S3–S5, ESI†). Each ZF domain has 28 amino acids, and sequence alignment revealed repetitive sequences, including CCHH and metal-coordination residues (Fig. 1b). Hydrophobic residues at the 12th Phe and 18th Leu positions in each ZF domain are conserved, thereby improving folding stability.^46,47^ Classical ZF domains are linked to the next ZF domains through TGEK(R)P; however, ZBTB20 lacks this conserved linker between its 4th and 5th ZF domains.^48^ The first four ZF domains are linked by seven amino acids, whereas the 5th ZF domain is connected by 25 amino acids (Fig. 1a). ZF domains are modular, allowing each domain in ZF proteins to function independently;^49^ therefore, we postulated that ZBTB20(ZF1-4) exhibits specific activity. To understand the oxidation pattern of ZBTB20, apo-ZBTB20(ZF1-4) was subjected to cobalt binding assays; however, d–d transitions were not observed because of rapid oxidation. The reported apo-PARIS(ZF2-4), a similar ZF domain, contains more than 90% reduced thiols when applied to Co^2+^.^19^ All experiments utilized the physiological metal ion Zn^2+^, and protein-interaction studies were conducted with Zn^2+^–ZBTB20(ZF1-4) or Zn^2+^–ZBTB20(ZF1-5).

### ZF domains from ZBTB20 for *afp* promoter regulation

**Fig. 2:**
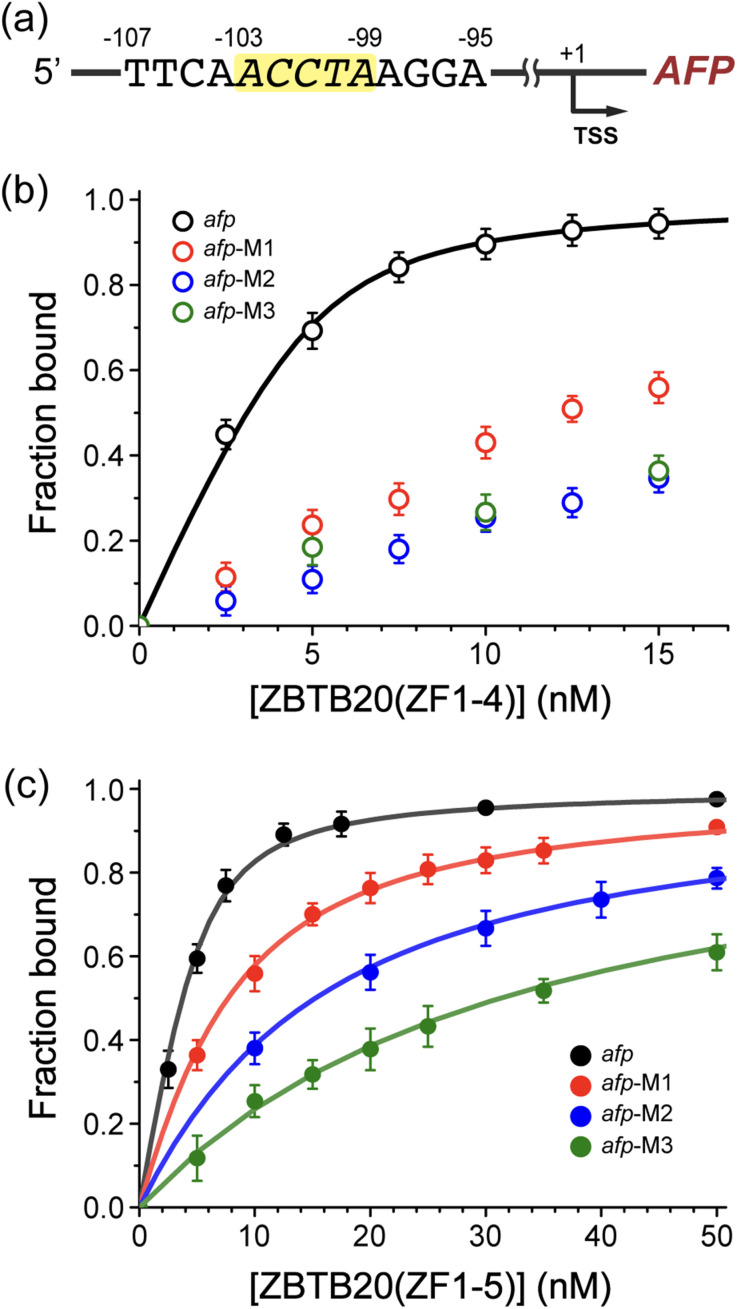
ZBTB20-mediated suppression of the alpha-fetoprotein (AFP). (a) Promoter region of *Homo sapiens afp* gene. The yellow highlighted sequence represents the core ZBTB20 binding site, located before the transcription start site (TSS). (b) and (c) Binding affinities of the wild-type (WT) *afp* promoter and its mutants with (b) ZBTB20(ZF1-4) and (c) ZBTB20(ZF1-5), measured *via* fluorescence anisotropy. Error bars represent standard deviations from a minimum of at least three independent experiments.

Previous studies have proposed that ZBTB20 suppresses the biological cascade involving AFP expression, although the specific biophysical mechanisms, including protein-binding domains, remain unreported.^23^ The putative *afp* promoter was identified as ACCTA (−103 to −99) positioned upstream of the transcriptional start site, as described in Fig. 2a and Fig. S6 (ESI†). To determine the binding affinity between ZBTB20 and the *afp* promoter region, fluorescence anisotropy (FA) was performed using purified ZBTB20 and a fluorescence-labeled 13-bp double-stranded DNA (Fig. 2b and c, ESI†). ZBTB20(ZF1-4) and ZBTB20(ZF1-5) have specific and selective binding affinities for the *afp* promoter, as indicated by the dissociation constants (*K*_d_s) shown in Table 1. The interaction of ZBTB20 (ZF1-4, *K*_d_ = 0.6 ± 0.04 nM) with the *afp* promoter was stronger than that of ZBTB20 (ZF1-5, *K*_d_ = 1.2 ± 0.08 nM), which indirectly indicated that the 5th ZF domain does not contribute to *afp* promoter binding. If the 5th ZF domain recognizes the same *afp* promoter sequence, the binding affinity would match that of ZBTB20(ZF1-4).

**Table 1:** Binding affinities about the interaction of ZBTB20-ZFs with *afp* promoter and mutants

Motif	DNA sequence	*K*_d_ (nM)	
		ZBTB20(ZF1-4)	ZBTB20(ZF1-5)
*afp*	5′-TTCAACCTAAGGA-F	0.6 (±0.04)	1.2 (±0.08)
*afp*-M1	5′-TTCA*G*CCTAAGGA-F	N/A	6.8 (±0.18)
*afp*-M2	5′-TTCA*G*CC*CC*AGGA-F	N/A	15.4 (±0.21)
*afp*-M3	5′-TTCAA*AAA*AAGGA-F	N/A	33.9 (±0.70)

Mutational studies of ZBTB20(ZF1-4) revealed its preference for specific sequences within the *afp* promoter, as indicated by anisotropy values that did not conform to proper curve-fitting (Fig. 2b). These results provide initial evidence that the ZF domains of ZBTB20 inhibit AFP expression in a sequence-sensitive manner. The binding affinity of ZBTB20(ZF1-4) was significantly decreased for single base pairs and other mutated base pairs, as shown in Fig. 2b and Table 1. According to mutational studies, the promoter sequence 5′-ACCTA is essential for the interaction between ZBTB20(ZF1-4). Both ZBTB20(ZF1-4) and ZBTB20(ZF1-5) are sensitive to the recognition of specific promoter sequences, with all five base pairs being crucial for *afp* suppression (Table 1). AFP expression must be tightly repressed, except during the prenatal period, and these strong interactions are vital in the liver.^50^ This study demonstrated that ZFs are the major binding domains for the *afp* promoter region located at −103 to −99, with remarkable selectivity (Fig. 2b).

### FOXO1 and its binding specificity to ZBTB20-ZFs

**Fig. 3:**
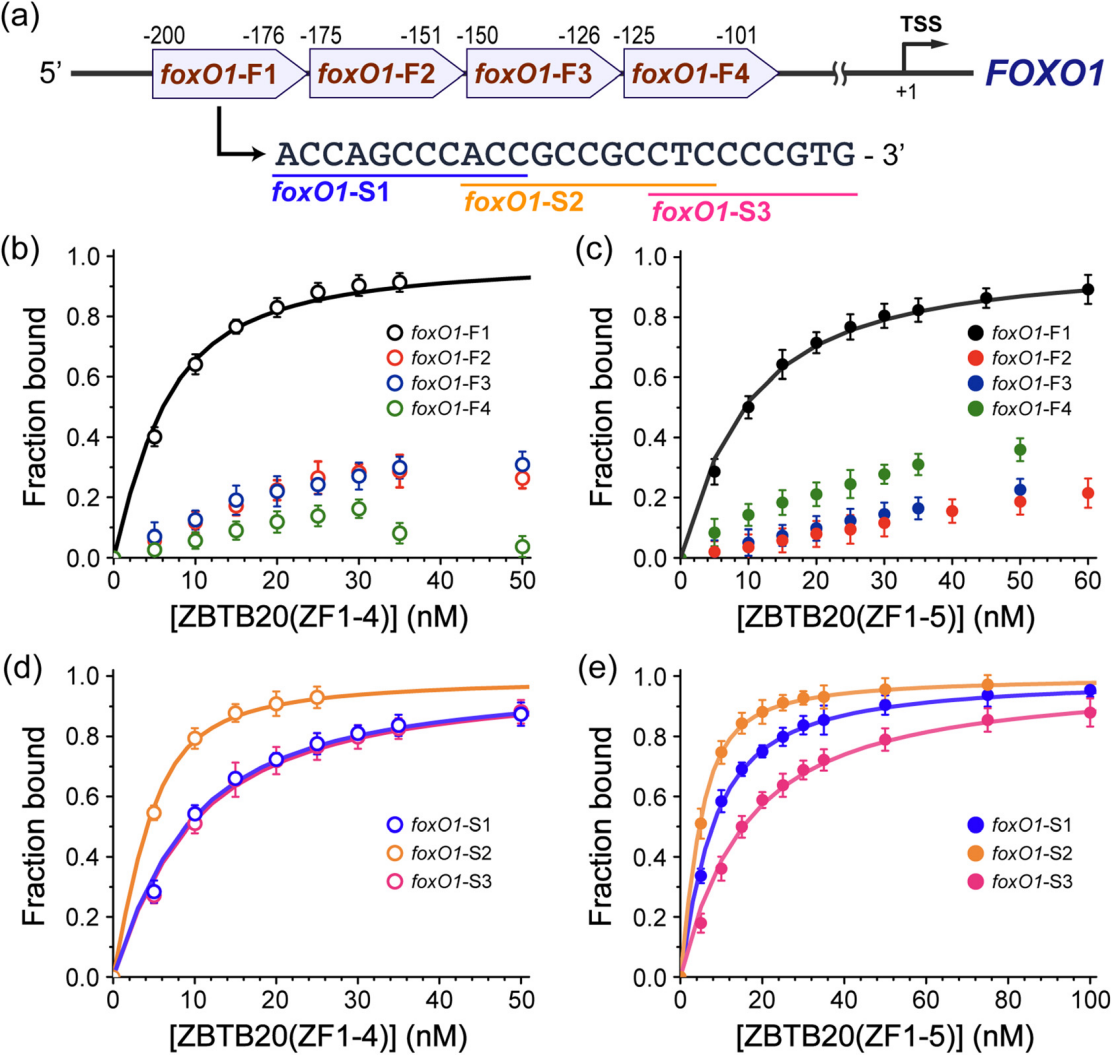
ZBTB20-mediated suppression of the forkhead box transcription factor O1 (FOXO1). (a) Putative binding site for ZBTB20 located upstream of *foxO1*. To identify specific binding sites for the ZBTB20 ZF domains, the *foxO1* promoter was divided into F1–F4 segments. The *foxO1*-F1 region was further divided into S1–S3 segments for a more detailed analysis, as shown below the promoter map. (b) and (c) Binding affinities of the *foxO1* promoter sections (F1–F4) with (b) ZBTB20(ZF1-4) and (c) ZBTB20(ZF1-5), measured *via* fluorescence anisotropy. (d) and (e) Binding affinities of the subdivided *foxO1*-F1 regions (S1–S3) with (d) ZBTB20(ZF1-4) and (e) ZBTB20(ZF1-5), respectively. The analysis identifies specific binding preferences within the *foxO1*-F1 region. Error bars indicate standard deviations from a minimum of three independent experiments.

FOXO1 arrests the cell cycle in response to DNA damage and inhibits cell proliferation.^38^ These roles of FOXO1 are crucial for HCC progression, and previous studies have suggested that FOXO1 can reverse the levels of ZBTB20.^6^ The binding affinity of FOXO1 was quantified using FA experiments involving ZF domains and the promoter elements of FOXO1 (Fig. 3). The putative promoter region of *foxO1* is located at the −200 to −100 region upstream,^33^ prompting us to segment the promoter region into four fragments from *foxO1*-F1 to *foxO1*-F4 (25 bp) to identify the interaction site (Fig. 3a and Fig. S7, ESI†). The results confirmed that ZBTB20(ZF1-4) and ZBTB20(ZF1-5) selectively bind to the F1 (−200 to −176) region with nanomolar (nM) *K*_d_s (Fig. 3b and c), and ZBTB20(ZF1-4) has a slightly higher affinity for *foxO1*-F1 than ZBTB20(ZF1-5) (Table 2). Furthermore, these two purified ZF domains displayed sequence specificity for *foxO1*-F1 elements, as other sequences, including *foxO1*-F2, -F3, and -F4 (−175 to −101 upstream of *foxO1*), could not determine *K*_d_s with ZBTB20 (Fig. 3b, c and Table 2).

**Table 2:** Binding affinities of ZBTB20-ZFs for fractions and segments of *foxO1* promoter

Motif	DNA sequence	*K*_d_ (nM)	
		ZBTB20(ZF1-4)	ZBTB20(ZF1-5)
*foxO1*-F1	5′-*AAA*ACCAGCCCACCGCCGCCTCCCCGTG*AAA*-F	3.6 (±0.20)	6.9 (±0.21)
*foxO1*-F2	5′-*AAA*GAAAACCGGGCCCCACCCAGCCCGG*AAA*-F	N/A	N/A
*foxO1*-F3	5′-*AAA*CGCCCACTGGCTGCCCGGGCGGCGG*AAA*-F	N/A	N/A
*foxO1*-F4	5′-*AAA*TGCCGCATGCCCATTGGCCGCGCGG*AAA*-F	N/A	N/A
*foxO1*-S1	5′-*AA*ACCAGCCCACC*AA*-F	6.4 (±0.26)	5.3 (±0.15)
*foxO1*-S2	5′-*AA*ACCGCCGCCTC*AA*-F	1.7 (±0.07)	2.1 (±0.05)
*foxO1*-S3	5′-*AA*CTCCCCGTG*AA*-F	6.8 (±0.31)	12.8 (±0.45)

Our findings confirmed the approximate location of the *cis*-acting element responsible for FOXO1 expression, which is the regulated *trans*-acting factor ZBTB20(ZF1-4) or ZBTB20(ZF1-5), although further specification is needed. The *foxO1*-F1 promoter was divided into three segments (S1–S3) to identify the binding sequences (Fig. 3a). Curve-fitting analysis showed that *foxO1*-S2 (−192 to −182, 5′-ACCGCCGCCTC) specifically bound to ZBTB20(ZF1-4) with *K*_d_ = 1.7 ± 0.07 nM, and this strong binding was also observed in ZBTB20(ZF1-5) with *K*_d_ = 2.1 ± 0.05 nM (Fig. 3d, e and Table 2). The ZF domains of ZBTB20 selectively attach to *cis*-acting elements to inhibit the activity of FOXO1, which can cause unexpected cell proliferation and block apoptosis.^35^ Although FOXO1 is regulated by ZBTB20, it was previously considered a super transcription factor.^6^ The etiology of HCC remains unclear; however, higher hierarchical transcriptional modulation can provide insights into combating this fatal disease. Although segment 2, *foxO1*-S2, exhibited selectivity for ZBTB20(ZF1-4 and ZF1-5), the other segments, *foxO1*-S1 and -S3, also demonstrated notable binding preferences. These remarkable interactions between *foxO1* and ZBTB20 can be attributed to upstream elements, including *foxO1*-S2 and also *foxO1*-S1 and -S3. FOXO1 must be tightly regulated to prevent deviation and proliferation during the cell cycle, whereas arrest requires stimuli and events that trigger the prompt action of FOXO1 with significant binding affinity.^51^

### Selectivity of ZBTB20 ZF domains for *cis*-acting elements from CX_2_CX_12_HX_3_H-type ZF domains and physiological metal ions

**Fig. 4:**
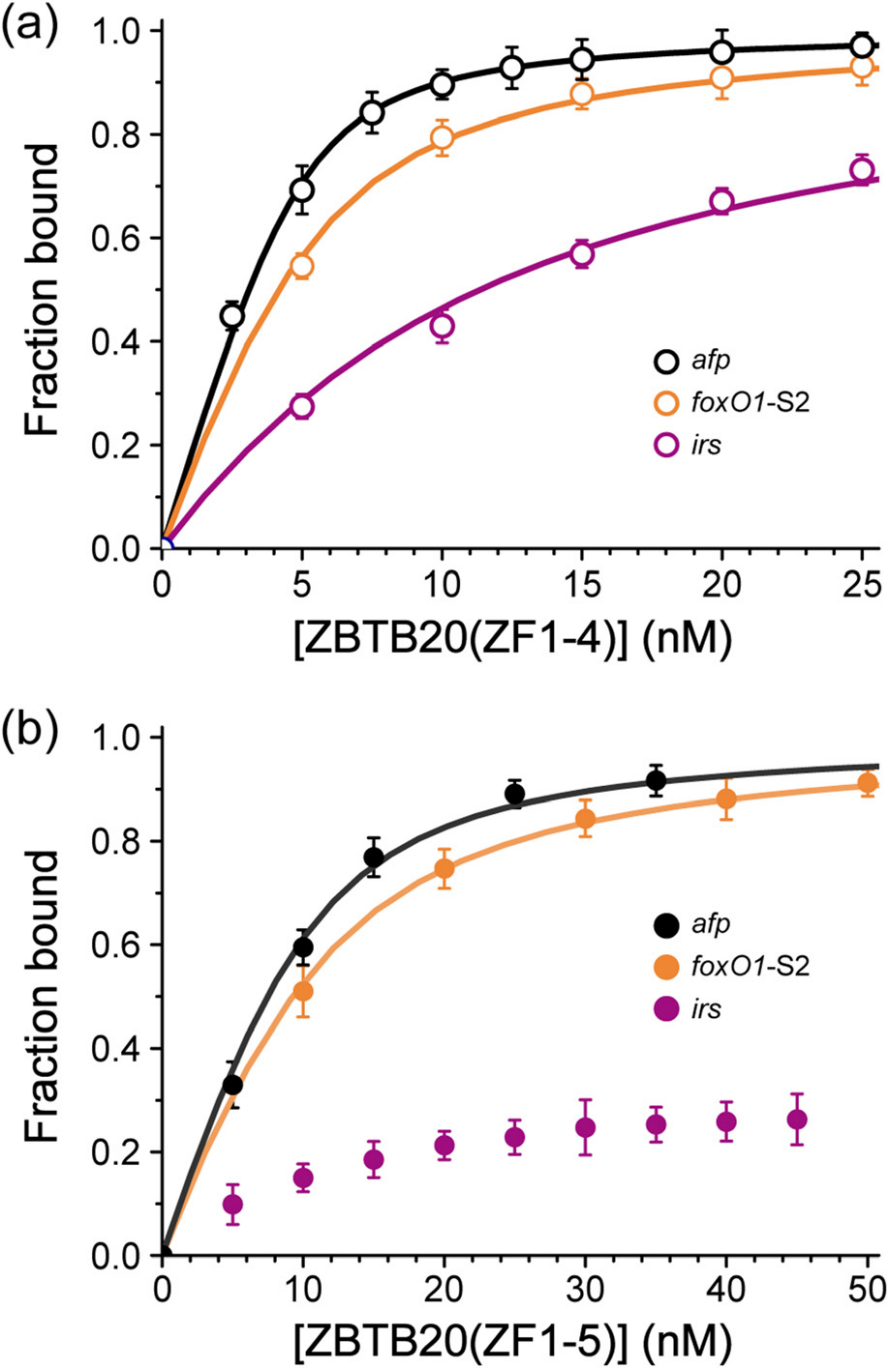
Transcriptional regulatory interactions of ZBTB20 with *cis*-acting elements. (a) and (b) Binding curve of *cis*-acting elements (*afp*, *foxO1*-S2, and *irs*) with (a) ZBTB20(ZF1-4) and (b) ZBTB20(ZF1-5), respectively. Error bars indicate standard deviations from at least three independent experiments.

Lee group suggested that ZF domains from PARIS, specifically PARIS(ZF2-4), selectively bind to insulin response sequence (IRS), identifying sequences (5′-TATTTTT) located upstream of peroxisome proliferator-activated receptor gamma coactivator 1α (PGC-1α), which subsequently inhibits dopamine release.^19^ The relationship between ZBTB20 and neuronal behaviors, including glioblastoma and astrocyte development in the brain, has been examined.^52,53^ Biophysical interactions were assessed using *irs*, revealing that ZBTB20(ZF1-4) binds strongly to the *irs* with *K*_d_ = 8.7 ± 0.67 nM, although not as strongly as *afp* and *foxO1* promoters (Fig. 4a and Table 3). In addition, ZBTB20(ZF1-5) did not exhibit sufficient interaction to allow for curve-fitting (Fig. 4b). The ZF domains of PARIS and ZBTB20 share the same CX_2_CX_12_HX_3_H-type ZF domains with high similarity (57%) and are both observed in the brain and other mammalian organs (Fig. S8, ESI†). Despite the similarity in ZF sequences, the oxidation rates and target promoter sequences differ considerably between PARIS and ZBTB20.^20^ The binding partner of *irs* is PARIS(ZF2-4) with a *K*_d_ of 38.9 ± 2.4 nM, indicating that ZBTB20(ZF1-4) binds tightly.^19^ These findings suggest that ZBTB20 may regulate signaling molecules in the brain. Notably, ZBTB20(ZF1-5) lacks specific and selective interactions with the *irs*, making it unfit for achieving *K*_d_ (Fig. 4b and Table 3). The functional role of the 5th ZF domain of ZBTB20, ZBTB20(ZF5), remains unclear, and further structural information is required to elucidate its binding affinity and interactions. The X-ray structure of transcription factor IIIA (TFIIIA), which binds to the 5S rRNA gene promoter (PDB: 1TF6), provides structural insights into the mechanisms of DNA interaction.^54^ The six ZF domains in TFIIIA exhibited distinct functional roles, with TFIIIA(ZF1-3) directly engaging the DNA major groove and TFIIIA(ZF4-6) contributing to structural stabilization or interacting with the DNA minor groove.^54^ Additionally, studies on ZNF217, a ZF protein with eight classical ZF domains, revealed that only specific ZF domains (ZF6 and ZF7) directly bind DNA, whereas other ZF domains perform regulatory or structural roles.^45^ This observation suggests that ZBTB20(ZF5) does not directly interact with DNA but instead plays a structural or regulatory role. ZBTB20(ZF5) is connected to ZF4 *via* an extended linker region, which may introduce steric hindrance or disrupt the ZBTB20(ZF1-4) alignment. ZBTB20(ZF5) might stabilize the structure or interact with the DNA minor groove like TFIIIA(ZF4-6) and potentially engage with proteins or nucleotides to modulate the conformation and flexibility of ZBTB20.^45,54^

**Table 3:** Comparison of binding affinities about ZBTB20-ZFs with *irs* and HCC-associated genes, *afp* and *foxO1*-S2

Motif	DNA sequence	*K*_d_ (nM)	
		ZBTB20 (ZF1-4)	ZBTB20 (ZF1-5)
*afp*	5′-TTCAACCTAAGGA-F	0.6 (±0.04)	1.2 (±0.08)
*foxO1*-S2	5′-*AA*ACCGCCGCCTC*AA*-F	1.7 (±0.07)	2.1 (±0.05)
*irs*	5′-GTGTTGGTATTTTTCCCTCAG- F	8.7 (±0.67)	N/A

The selectivity and binding affinity of ZF domains can be influenced by physiological factors, including the type and concentration of metal ions present in the cellular environment.^55^ Hepatic iron accumulation promotes HCC through mechanisms such as reactive oxygen species (ROS) generation, p53 suppression, induction of cell proliferation, and chronic inflammation.^56^ Our results showed that Fe^3+^ concentrations up to 100 nM had a subtle impact on the binding affinity between ZBTB20(ZF1-4) and *afp* or *foxO1* promoters, whereas higher concentrations (1.0 μM) resulted in a significant loss of affinity (Fig. S9, ESI†). This suggests that hepatic iron accumulation may modulate the gene-specific binding behavior of ZBTB20(ZF1-4).

## Conclusions

Transcription factors initiate physiological responses to biological stimuli within our systems, with ZF proteins facilitating the initial interactions that trigger signaling cascades.^57^ Bioinformatic studies suggest that over 3% of the human genome encodes ZF proteins, although the majority require experimental validation.^58,59^ As classical ZFs, CX_2_CX_12_HX_3_H-type ZF proteins are ubiquitously observed, including in the brain, and studies have demonstrated that PARIS plays a crucial role in regulating dopamine release in the neuronal system.^18^ According to the Kyoto Encyclopedia of Genes and Genomes (KEGG), ZBTB20 is part of the central network in cancer regulation, although further biophysical studies are necessary.^51^ Our study targeted two transcription factors, AFP and FOXO1, and examined their biophysical properties using purified ZF domains. The promoter region that induces protein expression was identified using purified ZF domains. Our initial findings revealed that ZBTB20(ZF1-4) inhibits AFP and FOXO1 expression. The determined *K*_d_s indicate that the protein-nucleic acid interactions are specific and selective.

We documented the suppression of dopamine release through the interactions between the ZF domains of PARIS and IRS,^19^ whereas the ZF domains of ZBTB20 do not induce specific binding to *irs*. Despite belonging to the superfamily of classical ZF domains, these two ZF domains exhibit several interesting characteristics. PARIS has four ZF domains, including one non-classical ZF domain, whereas ZBTB20 has five classical ZF domains.^18^ Three classical domains from PARIS demonstrated specific interactions with target nucleic acids,^20^ and four classical ZF domains from ZBTB20 induced strong interactions with its cognitive DNA for more than five ZF domains. These findings confirm that ZF domains function independently within ZF proteins to regulate protein expression. In addition, the domains of ZBTB20 and PARIS exhibit different oxidation rates, which are crucial for metal coordination. PARIS shows more than 90% of reduced domains after purification,^19^ whereas ZBTB20 is completely oxidized after apo-protein generation. The rapid oxidation of these ZF domains may pose a challenge for their application in biotechnology, and the primary structures of these ZF domains can provide crucial information.^59,60^

**Fig. 5:**
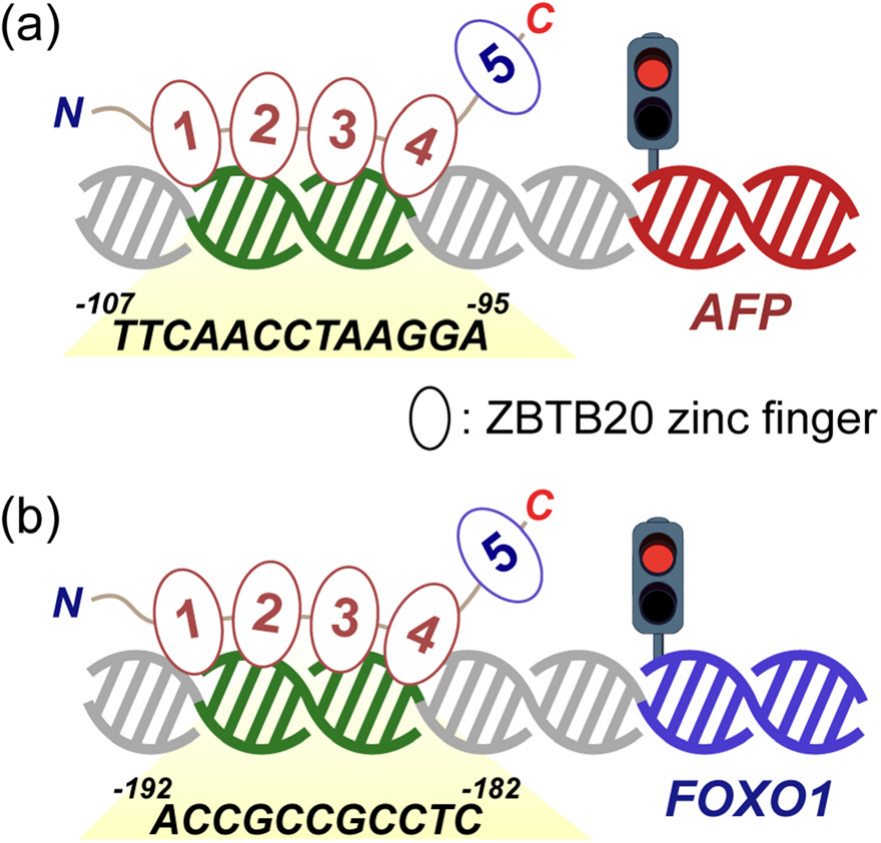
Schematic of ZBTB20 zinc finger (ZF) binding to *cis*-acting elements in cancer regulation. (a) and (b) ZBTB20 ZF domains bound to (a) *afp* promoter contributing to tumor suppression, and (b) *foxO1* promoter facilitating tumorigenesis.

This study highlights the crucial role of transcription factor expression in maintaining homeostasis in biological systems (Fig. 5).^23,33^ The dual functions of these two proteins should be meticulously controlled to avoid unwanted side effects that can lead to severe carcinogenesis in the brain and other organs of the body. To determine the appropriate expression systems, the cancer-inducing effects in various organs should be investigated using these two models. The molecular mechanisms and interactions of ZBTB20 within cellular systems should be further explored to better understand its central role in cancer regulation and its potential as a therapeutic target.

## Experimental section

### Chemicals

Chemicals required to perform various experiments were purchased and used to express ZBTB20(ZF1-4) and ZBTB20(ZF1-5). Luria-Bertani broth (LB, Duchefa Biochemie), ampicillin sodium (Goldbio), and isopropyl-β-d-1-thiogalactopyranoside (IPTG, Goldbio) were used for stimulating cell growth and protein expression. Purification was performed using SP-sepharose (Cytiva) and Superdex75 (Cytiva) coupled with an ÄKTA Pure 25 L (Cytiva). The following chemicals were used for the purification process: 3-(*N*-morpholino) propanesulfonic acid (MOPS, Fisher bioreagents), sodium chloride (NaCl, DAEJUNG), magnesium chloride anhydrous (MgCl_2_, DAEJUNG), dithiothreitol (DTT, GoldBio), glycerol (Junsei), DNase (Takara), zinc sulfate heptahydrate (Sigma-Aldrich), and phenylmethylsulfonyl fluoride (PMSF, Thermo Scientific). The supernatant was filtered using an ultracentrifuge (Hanil Science and BECKMAN) and the concentration of purified ZF domains was measured using a UV-Vis spectrophotometer (Agilent Technologies, Cary 60) in a cuvette (Hellma Analytics).

### Expression of ZBTB20(ZF1-4) and ZBTB20(ZF1-5)

Codon optimization of the *Homo sapiens zbtb20* nucleotide sequence (NCBI: BC029041.1) was requested from BIONICS. The codon-optimized *zbtb20(zf1-4)* and *zbtb20(zf1-5)* genes were synthesized and cloned into the *pET-30a(+)* vector, resulting in the constructs *zbtb20(zf1-4)-pET-30a(+)* and *zbtb20(zf1-5)-pET-30a(+)*, respectively. The *zbtb20(zf1-4)-pET-30a(+)* construct was transformed into Rosetta(DE3) cells to express ZBTB20(ZF1-4), whereas *zbtb20(zf1-5)-pET-30a(+)* was transformed into HIT(DE3) cells for the expression of ZBTB20(ZF1-5). The transformed cells were cultured in 200 mL LB broth containing kanamycin at 37 °C and shaken at 200 rpm for 12–16 h. The expression of ZBTB20(ZF1-4) and ZBTB20(ZF1-5) was induced using IPTG (0.1 mM) with zinc sulfate heptahydrate (0.1 mM) at 25 °C for 7 h and 20 °C for 8 h, respectively. Cell debris was pelleted by centrifugation at 11 355 × *g* at 4 °C for 20 min. The cell pellets were lysed in buffer A (composed of 25 mM MOPS, 50 mM NaCl, 5 mM MgCl_2_, 1 mM DTT, 0.01 μL mL^−1^ DNaseI, and 0.002 mg mL^−1^ PMSF; pH 7.5) using a sonicator (Sonics) for 40 min (on for 15 s and off for 45 s). The entire cell lysate was centrifuged at 28 306 × *g* at 4 °C and filtered using a 0.22 μm membrane syringe filter to obtain the supernatant.

### Purification of ZBTB20(ZF1-4) and ZBTB20(ZF1-5)

The supernatant was introduced into an SP-sepharose column, rinsed with activating buffer B (composed of 25 mM MOPS, 50 mM NaCl, 1 mM DTT, and 5% glycerol; pH 7.4), and eluted using a linear gradient ranging from 50 to 1000 mM NaCl. The presence of ZBTB20(ZF1-4) and ZBTB20(ZF1-5) was confirmed by 15% sodium dodecyl sulfate–polyacrylamide electrophoresis (SDS–PAGE) followed by Coomassie brilliant blue staining. Fractions containing the target proteins were concentrated at 4 °C and 2095 × *g* using a 10 kDa cut-off membrane filter (Merck Millipore). The concentrated proteins were further purified by loading onto a Superdex 75 column equilibrated with activating buffer B. Finally, the samples were reconcentrated using a 10 kDa cut-off membrane filter at 4 °C and 2095 × *g*. The concentrated eluate was subsequently quantified *via* UV-Vis spectrometry, with extinction coefficients (*ε*_280_) of 14 940 M^−1^ cm^−1^ for ZBTB20(ZF1-4) and 16 555 M^−1^ cm^−1^ for ZBTB20(ZF1-5), and stored at −88 °C. The molecular weights of ZBTB20(ZF1-4) (13 913.21 Da) and ZBTB20(ZF1-5) (19 196.16 Da) were determined using SDS–PAGE.

### Measurement of fluorescence anisotropy

Fluorescence anisotropy was measured using an FP8300 spectrofluorometer (JASCO) with cuvettes (JASCO, J/3 type). The 3′-end of the DNA was labeled with 6-carboxyfluorescein (6-FAM) fluorescein dye (Integrated DNA Technology; IDT). The excitation and emission absorbances were measured at 499 and 518 nm, respectively. All measurements were performed under the following conditions: bandwidth, 5 nm; response, 0.5 s; and PMT voltage of 700 V at 25 °C. The DNA, tagged with fluorescence at a concentration of 5 nM DNA in 1600 μL of buffer C (composed of 25 mM MOPS, 50 mM NaCl, 1 mM DTT, and pH 7.4), was titrated by adding proteins, and the anisotropy was measured thrice following 2-min reactions. Dissociation constants for the interaction between protein and DNA were obtained using the 1 : 1 binding model. The overall calculations were performed using the following equations:
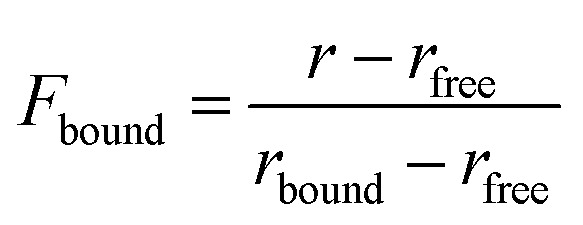
where *F*_bound_ (fraction bound) indicates the fraction of DNA-bound protein, *r*_free_ indicates the anisotropy of unbound DNA, and *r*_bound_ indicates the anisotropy of protein-bound DNA when saturated. The dissociation constant (*K*_d_s) was measured using the 1 : 1 binding model based on the following equation:^19^

*P* + *D* ⇌ *PD*
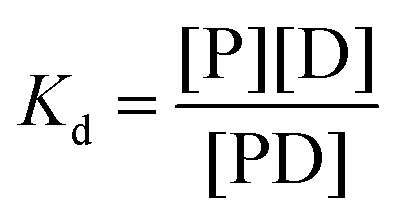

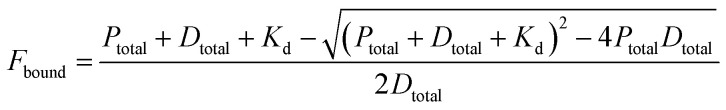
where *P* indicates the protein concentration and *D* indicates the DNA concentration.

## Author contributions

Hyunyong Kim: conceptualization; methodology; investigation, formal analysis; writing – original draft and review and editing. Yunha Hwang: conceptualization; methodology; investigation, formal analysis; validation; writing – original draft and review and editing. Jin Sung Cheong: conceptualization, methodology, formal analysis, validation, writing – review and editing. Seung Jae Lee: funding acquisition; project administration; supervision; conceptualization; investigation; methodology; writing – original draft; writing – review and editing methodology, validation, and resources.

## Data availability

The data supporting this article have been included as part of the ESI.† The data are plotted with the Origin software.

## Conflicts of interest

The authors declare that they do not have any conflicts of interest.

## Supplementary Material

CB-OLF-D5CB00088B-s001
