# Supplementary material for: Regulation of carcinogenesis through multiple zinc fingers in ZBTB20
Source: RSC Chem Biol. 2025 Jun 9;6(7):1165–73. doi: 10.1039/d5cb00088b (PMC12146892; doi:10.1039/d5cb00088b)
Supplement: CB-006-D5CB00088B-s001 [file CB-006-D5CB00088B-s001.pdf]

Supplementary Information (SI) for RSC Chemical Biology.
This journal is © The Royal Society of Chemistry 2025

Supplementary Information

for

Regulation of carcinogenesis through multiple zinc fingers in ZBTB20

Hyunyong Kim,[a]† Yunha Hwang,[a]† Jin Sung Cheong,[b] and Seung Jae Lee*[a,c]

[a]Department of Chemistry, Jeonbuk National University, Jeonju 54896, Republic of Korea

[b]Department of Neurology, Wonkwang University Hospital, Iksan 54538, Republic of Korea

[c]Institute for Molecular Biology and Genetics, Jeonbuk National University, Jeonju 54896, Republic of Korea

†These authors contributed equally to this work

*To whom correspondence should be addressed:

Seung Jae Lee, Ph.D.

Professor,

Department of Chemistry and Institute for Molecular Biology and Genetics,

Jeonbuk National University, Jeonju 54896, Rep. of Korea

E-mail: slee026@jbnu.ac.kr

Tel. +82-63-270-3412

Fax. +82-63-270-3407

Table of Contents

**Figure S1**	Amino acid sequence of *Homo sapiens* ZBTB20	S3
**Figure S2**	Structure of transcriptional regulator in tumorigenesis	S4
**Figure S3**	Codon-optimization of *Homo sapiens* ZBTB20(ZF1-5)	S5
**Figure S4**	Purification and characterization of *Homo sapiens* ZBTB20(ZF1-4)	S6
**Figure S5**	Purification and characterization of *Homo sapiens* ZBTB20(ZF1-5)	S7
**Figure S6**	Promoter region of *Homo sapiens afp*	S8
**Figure S7**	Promoter region of *Homo sapiens foxO1*	S9
**Figure S8**	Comparison of ZBTB20 and PARIS	S10
**Figure S9**	Interaction of ZBTB20(ZF1-4) with HCC-associated genes under Fe^{3+} conditions	S11

References S12

BTB/POZ domain

1 MTERIHSINL HNFSNSVLET LNEQRNRGHF CDVTVRIHGS MLRAHRCVLA AGSPFFQDKL 60

61 LLGYSDIEIP SVVSVQSVQK LIDFMYSGVL RVSQSEALQI LTAASILQIK TVIDECTRIV 120

121 SQNVGDVFPG IQDSGQDTPR GTPESGTSGQ SSDTESGYLQ SHPQHSVDRI YSALYACSMQ 180

181 NGSGERSFYS GAVVSHHETA LGLPRDHHME DPSWITRIHE RSQQMERYLS TTPETTHCRK 240

241 QPRPVRIQTL VGNIHIKQEM EDDYDYYGQQ RVQILERNES EECTEDTDQA EGTESEPKGE 300

301 SFDSGVSSSI GTEPDSVEQQ FGPGAARDSQ AEPTQPEQAA EAPAEGGPQT NQLETGASSP 360

361 ERSNEVEMDS TVITVSNSSD KSVLQQPSVN TSIGQPLPST QLYLRQTETL TSNLRMPLTL 420

421 TSNTQVIGTA GNTYLPALFT TQPAGSGPKP FLFSLPQPLA GQQTQFVTVF QPGLSTFTAQ 480

ZF1 ZF2

481 LPAPQPLASS AGHSTASGQG EKKPYECTLC NKTFTAKQNY VKHMFVHTGE KPHQCSICWR 540

ZF3 ZF4

541 SFSLKDYLIK HMVTHTGVRA YQCSICNKRF TQKSSLNVHM RLHRGEKSYE CYICKKKFSH 600

ZF5

601 KTLLERHVAL HSASNGTPPA GTPPGARAGP PGVVACTEGT TYVCSVCPAK FDQIEQFNDH 660

661 MRMHVSDG 668

Figure S1. Amino acid sequence of *Homo sapiens* ZBTB20. (GenBank: AAH29041.1)

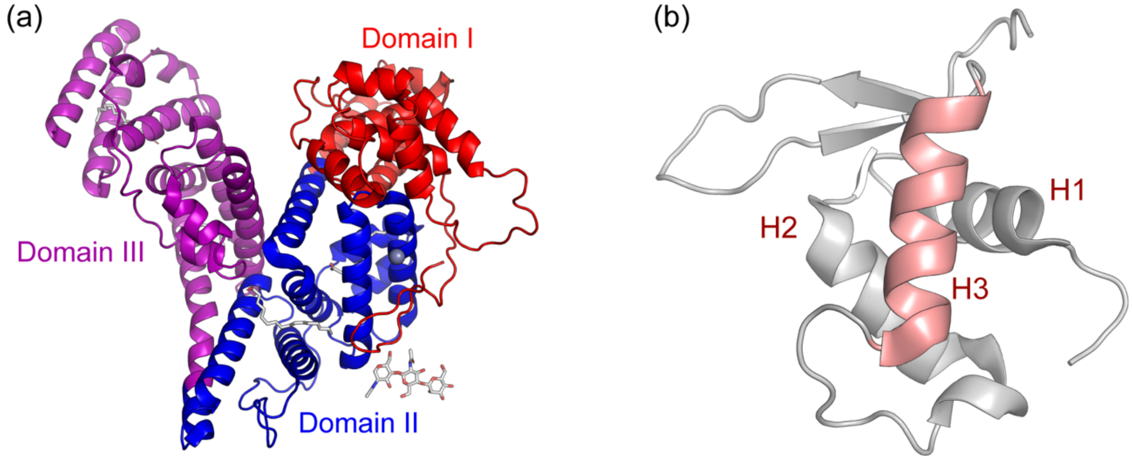

Figure S2. Structure of transcriptional regulators in tumorigenesis. (a) Cryo-EM structure of *Homo sapiens* AFP (PDB code: 8X1N).[1] (b) X-ray structure of *Homo sapiens* FOXO1 (PDB code: 5DUI).[2] Helices 1 and 2 are shown in gray, whereas helix 3 and the DNA-binding domain (FOXO1-DBD) are highlighted in pink.

```
GAA AAA AAG CCT TAT GAG TGC ACT CTC TGC AAC AAG ACT TTC ACC GCC AAA CAG AAC TAC
GAG AAA AAA CCG TAT GAA TGC ACC CTG TGC AAC AAA ACG TTT ACC GCG AAA CAG AAC TAT
 E   K   K   P   Y   E   C   T   L   C   N   K   T   F   T   A   K   Q   N   Y

GTC AAG CAC ATG TTC GTA CAC ACA GGT GAG AAG CCC CAC CAA TGC AGC ATC TGT TGG CGC
GTG AAA CAC ATG TTT GTT CAC ACC GGT GAA AAA CCG CAC CAG TGC AGC ATC TGC TGG CGT
 V   K   H   M   F   V   H   T   G   E   K   P   H   Q   C   S   I   C   W   R

TCC TTC TCC TTA AAG GAT TAC CTT ATC AAG CAC ATG GTG ACA CAC ACA GGA GTG AGG GCA
AGC TTC TCT CTG AAA GAC TAC CTG ATC AAA CAC ATG GTG ACC CAC ACC GGT GTT CGC GCT
 S   F   S   L   K   D   Y   L   I   K   H   M   V   T   H   T   G   V   R   A

TAC CAG TGT AGT ATC TGC AAC AAG CGC TTC ACC CAG AAG AGC TCC CTC AAC GTG CAC ATG
TAC CAG TGC TCT ATC TGC AAC AAG CGC TTC ACC CAA AAA TCC TCT CTG AAC GTT CAC ATG
 Y   Q   C   S   I   C   N   K   R   F   T   Q   K   S   S   L   N   V   H   M

CGC CTC CAC CGG GGA GAG AAG TCC TAC GAG TGC TAC ATC TGC AAA AAG AAG TTC TCT CAC
CGC CTG CAC CGC GGT GAA AAA TCT TAT GAA TGC TAC ATT TGC AAA AAG AAA TTC TCT CAC
 R   L   H   R   G   E   K   S   Y   E   C   Y   I   C   K   K   K   F   S   H

AAG ACC CTC CTG GAG CGA CAC GTG GCC CTG CAC AGT GCC AGC AAT GGG ACC CCC CCT GCA
AAA ACC CTG CTG GAA CGT CAT GTG GCG CTG CAC AGC GCG AGC AAC GGT ACC CCG CCG GCG
 K   T   L   L   E   R   H   V   A   L   H   S   A   S   N   G   T   P   P   A

GGC ACA CCC CCA GGT GCC CGC GCT GGC CCC CCA GGC GTG GTG GCC TGC ACG GAG GGG ACC
GGC ACC CCG CCG GGT GCG CGT GCG GGT CCG CCG GGT GTT GTG GCA TGT ACT GAA GGT ACC
 G   T   P   P   G   A   R   A   G   P   P   G   V   V   A   C   T   E   G   T

ACT TAC GTC TGC TCC GTC TGC CCA GCA AAG TTT GAC CAA ATC GAG CAG TTC AAC GAC CAC
ACC TAC GTT TGC TCC GTG TGC CCG GCA AAA TTT GAT CAG ATT GAA CAG TTC AAC GAT CAC
 T   Y   V   C   S   V   C   P   A   K   F   D   Q   I   E   Q   F   N   D   H

ATG AGG ATG CAT GTG TCT GAC GGA TAA
ATG CGT ATG CAC GTT AGC GAT GGT TAA
 M   R   M   H   V   S   D   G
```

Figure S3. Codon optimization of *Homo sapiens* ZBTB20(ZF1-5). The black DNA sequence represents the original sequence, and the purple DNA sequence indicates the codon-optimized sequence. The bold sequences correspond to amino acids encoded by each codon.

(a) 1MEKKPYECTL CNKTFTAKQN YVKHMFVHTG EKPHQCSICW RSFSLKDYLI KHMVTHTGVR60
61AYQCSICNKR FTQKSSLNVH MRLHRGEKSY ECYICKKKFS HKTLLERHVA LHSASNGT118

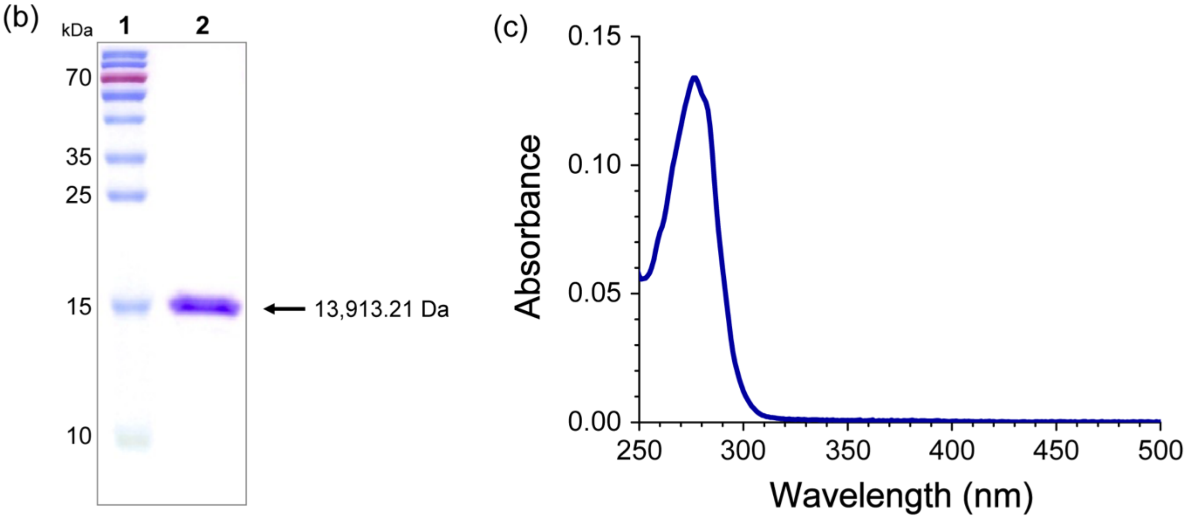

Figure S4. Purification and characterization of *Homo sapiens* ZBTB20(ZF1-4). (a) Amino acid sequence of ZBTB20(ZF1-4). (b) SDS-PAGE analysis of ZBTB20(ZF1-4). Lanes 1 and 2 indicate molecular marker and purified ZBTB20(ZF1-4), respectively. (c) UV-Vis spectrum of ZBTB20(ZF1-4).

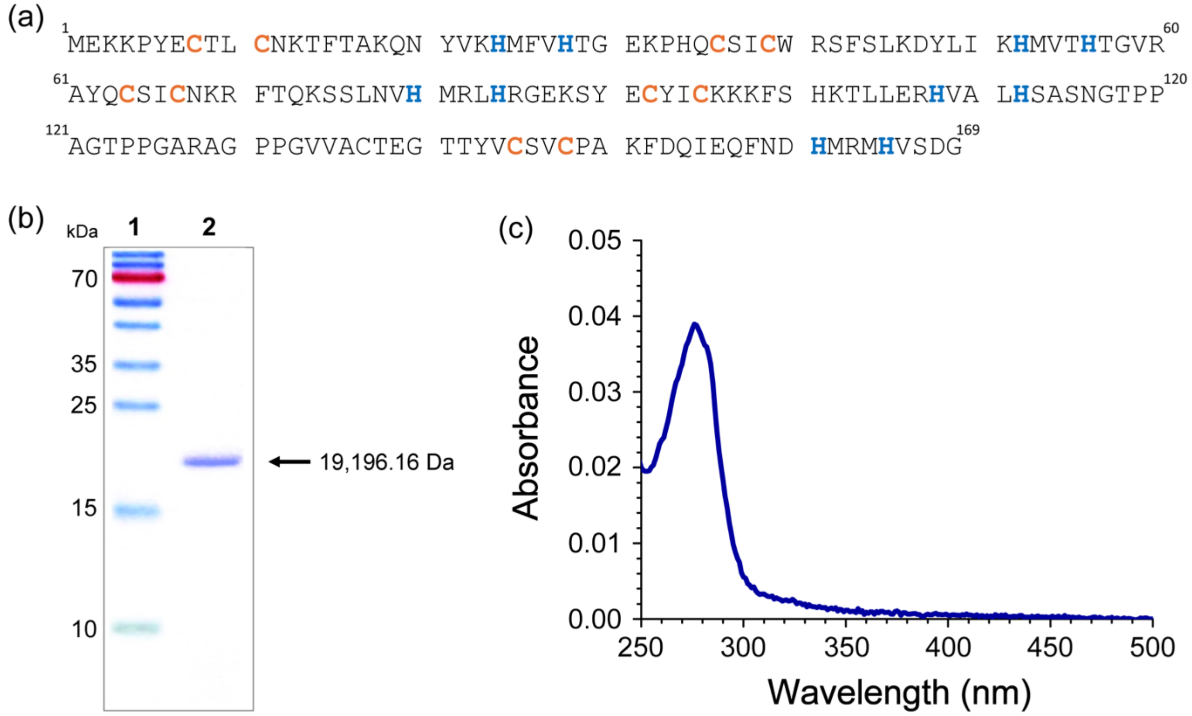

Figure S5. Purification and characterization of *Homo sapiens* ZBTB20(ZF1-5). (a) Amino acid sequence of ZBTB20(ZF1-5). (b) SDS-PAGE analysis of ZBTB20(ZF1-5). Lanes 1 and 2 indicate molecular marker and purified ZBTB20(ZF1-5), respectively. (c) UV-Vis spectrum of ZBTB20(ZF1-5).

-107 -95

AAATGTCCCATTTTCAACCTAAGGAAATACCATAAAGTAACAGATATACCAACAAAAGGTTAC

+1

TAGTTAACAGGCATTGCCTGAAAAGAGTATAAAAGAATTTCAGCATGATTTTCCATATTGTGC

TSS

TTCCACCACTGCCAATAACAAAATAACTAGCAACC ATG AAG TGG GTG GAA TCA ATT

AFP →

Figure S6. Promoter region of *Homo sapiens afp* (NCBI: NC_000004.12)[3]

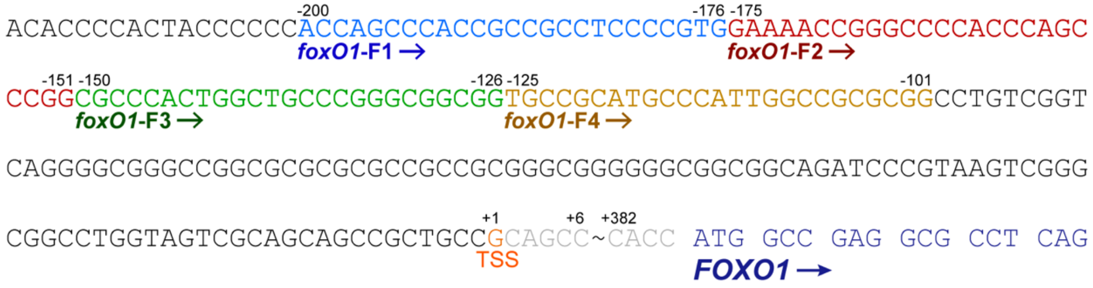

Figure S7. Promoter region of *Homo sapiens foxO1* (NCBI: NC_060937.1)[4]

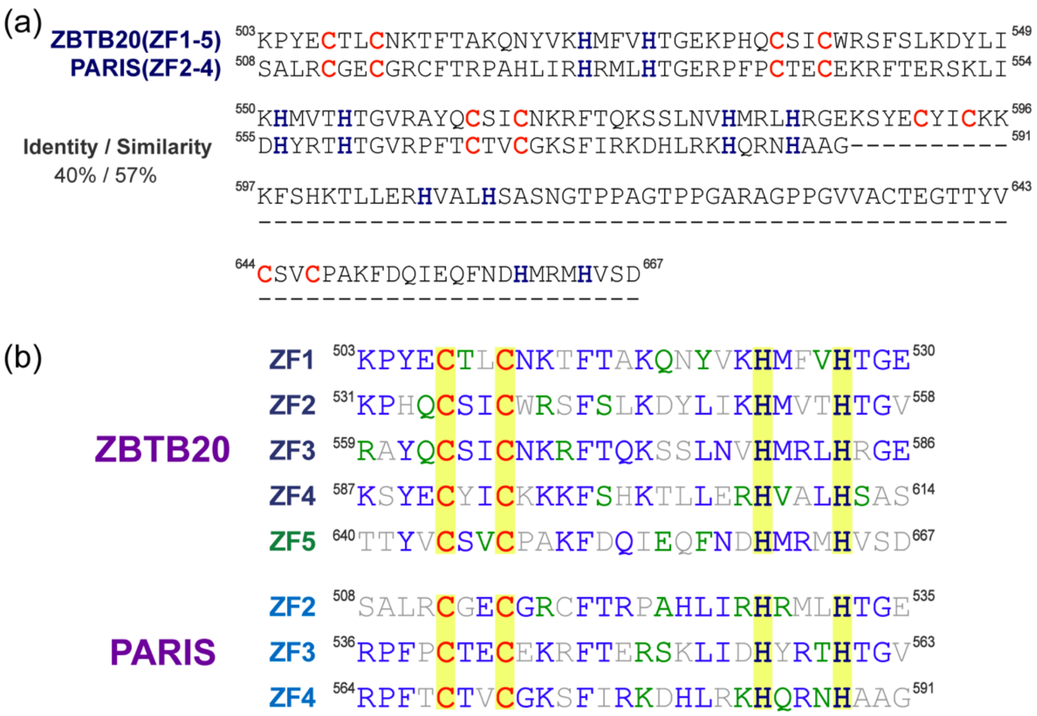

Figure S8. Comparison of ZBTB20 and PARIS. (a) Sequence alignment of *Homo sapiens* ZBTB20(ZF1-5) (NCBI: AAH29041.1) with *Homo sapiens* PARIS(ZF2-4) (NCBI: Q6NUN9.1).[5] (b) Multiple sequence alignment of conserved residues in ZBTB20(ZF1-5) and PARIS(ZF2-4). Each color represents an identity (blue), similarity (green), and mismatch (gray) sequence. Yellow highlights indicate cysteine (Cys) and histidine (His) residues involved in zinc ion coordination, characteristic of the $CX_2CX_{12}HX_3H$-type zinc finger motif.

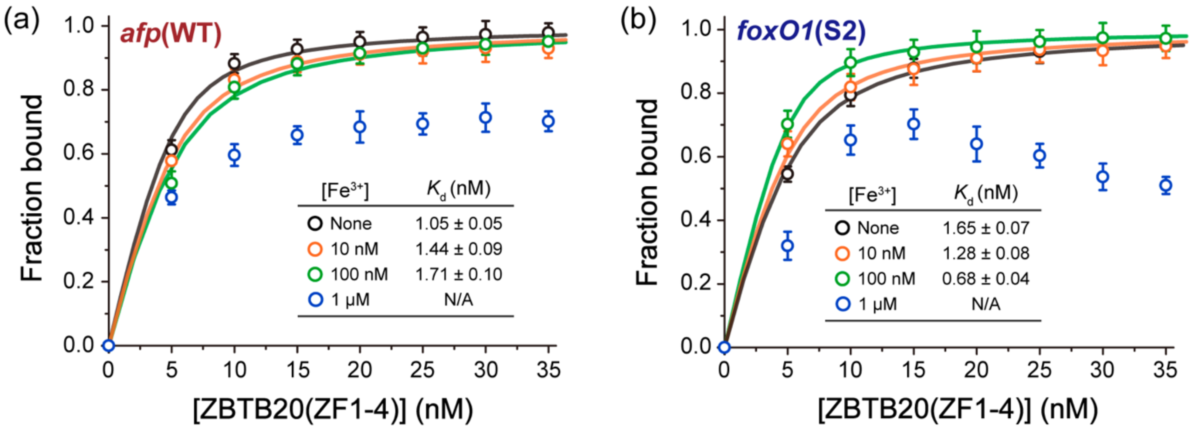

Figure S9. Interaction of ZBTB20(ZF1-4) with HCC-associated genes under Fe^{3+} conditions. The binding affinity of ZBTB20(ZF1-4) was measured under varying Fe^{3+} concentrations for (a) the *afp* promoter and (b) the *foxO1*-S2 promoter. Conditions: [DNA] = 5 nM; 25 mM MOPS (pH 7.4), 50 mM NaCl, 1 mM TCEP, (0, 10, 100 nM, and 1.0 μM) $FeCl_3 \cdot 6H_2O$; 25 °C.

References

1 K. Liu, C. Wu, M. Zhu, J. Xu, B. Lin, H. Lin, Z. Liu and M. Li, *Commun. Biol.*, 2024, **7**, 505.

2 P. Singh, E. H. Han, J. A. Endrizzi, R. M. O'Brien and Y.-I. Chi, *J. Struct. Biol.*, 2017, **198**, 54-64.

3 H. Zhang, D. Cao, L. Zhou, Y. Zhang, X. Guo, H. Li, Y. Chen, B. T. Spear, J.-W. Wu and Z. Xie, *Sci. Rep.*, 2015, **5**, 11979.

4 H. Kan, Y. Huang, X. Li, D. Liu, J. Chen and M. Shu, *Oncotarget*, 2016, **7**, 14336.

5 Y. Hwang, A. K. Mohammad Mydul Islam, S. Park, H. G. Kang, C. Lee, M. H. Lim and S. J. Lee, *ACS Chem. Neurosci.*, 2024, **15**, 447-455.
